# Eight quick tips for including chromosome X in genome-wide association studies

**DOI:** 10.1371/journal.pcbi.1012160

**Published:** 2024-06-06

**Authors:** Justin Bellavance, Linda Wang, Sarah A. Gagliano Taliun

**Affiliations:** 1 Faculty of Medicine, Université de Montréal, Montréal, Québec, Canada; 2 Research Centre, Montréal Heart Institute, Montréal, Québec, Canada; 3 Department of Medicine, Faculty of Medicine, Université de Montréal, Montréal, Québec, Canada; 4 Department of Neurosciences, Faculty of Medicine, Université de Montréal, Montréal, Québec, Canada; SIB Swiss Institute of Bioinformatics, SWITZERLAND

## Introduction

All individuals carry a minimum of 1 copy of chromosome X. Despite being a relatively long chromosome with more than 150 million base pairs [1], similar in length to chromosome 8, association testing of genetic variants on chromosome X is still not routinely conducted. Genome-wide association studies (GWAS) have been used to identify a vast range of genomic loci of interest for a variety of complex human diseases and traits by quantifying genetic variants that are statistically associated with a given disease/trait [2,3]. However, a lack of testing for variants on the X chromosome limits our ability to identify vital loci and subsequently understand potential mechanisms linked to this chromosome. There was a call for the inclusion of chromosome X into genome-wide association analyses presented in 2013. At that time, a scan of published GWAS from 2010 and 2011 showed that only 33% of the studies had tested variants on the X chromosome in their analyses [4]. Despite this call for inclusion, the lack of representation of this chromosome has not improved according to a 2023 study. Of the 136 publications that submitted at least 1 summary statistics file to the NHGRI-EBI GWAS Catalog in 2021, only 25% reported chromosome X results [5].

Indeed, there are several characteristics of this chromosome that make it unique compared to the autosomes, which can pose analytical challenges in association testing. Such challenges include how to account for X inactivation in individuals with an XX karyotype, how to model the hemizygous state of genotypes in individuals with an XY karyotype, or how to best code genotypes at the 2 pseudo-autosomal regions, short stretches at either end of the X with high homology with the Y chromosome, known as PAR1 and PAR2. The non-pseudo-autosomal region (nonPAR) denotes the middle sequence of the X chromosome.

Furthermore, there are many well-used software that take GWAS summary statistics as input and ignore chromosome X information [6,7]. This practice can make it difficult and unintuitive for researchers to run association testing on the X chromosome.

Inclusion of chromosome X routinely in GWAS and downstream analyses will serve to enhance our understanding of the genetic contributors to complex diseases and traits. Here, we propose 8 tips to help move towards the inclusion of X in GWAS to provide a suggested set of concrete actions that can be taken to overcome the challenges or obstacles preventing routine analysis of this chromosome.

## Tip 1: Retain chromosome X variants in your quality control pipeline

Chromosome X variants tend to have lower quality whether genotyped, sequenced, or imputed compared to autosomal variants [8,9]. Perform careful quality control checks genome-wide, including variants on the X chromosome instead of discarding genotyping or sequencing calls for variants on the X chromosome. For sequencing data, depth on the X can be used to get a sense of each study participant’s sex chromosome karyotype. For genotyping array data, F statistics of X chromosome heterozygosity, as implemented in PLINK, for example, can be estimated to accomplish this task. Chromosome X-specific variant filters as reviewed by Keur and colleagues [10] should be incorporated, including testing for missingness by sex, call rate by sex, and the proportion of heterozygotes in individuals with XY karyotypes should be assessed. Additionally, Hardy–Weinberg Equilibrium models accounting for different ploidy on chromosome X (for example, diploid for females and haploid for males in the nonPAR region) with different assumptions with regard to X inactivation (see Tip 6) have been proposed [11].

## Tip 2: Impute chromosome X variants

For genotyping array data, maximize the number of genetic variants available for association testing by imputing genotypes of variants that are not present on the array, including variants on the X chromosome. Various free online resources can be used to carry out the computationally intensive task of genotype imputation using available whole-genome sequencing imputation panels (**Table 1**), including the Trans-Omics for Precision Medicine (TOPMed) Imputation Server [12], Sanger Imputation Service [13], and Michigan Imputation Server [14]. The TOPMed and Michigan Imputation Servers will perform some basic chromosome X-specific quality control on the uploaded genotypes, including verifying that all variants in the nonPAR region are either haploid or diploid. For these 2 servers, chromosome X is split into 3 parts (PAR1, nonPAR, and PAR2) for statistical phasing and genotype imputation, and then subsequently merged. In contrast, the Sanger Imputation Service does not perform quality control steps on the genotypes. A thorough assessment of the various imputation panels and tools available to handle chromosome X imputation is pertinent but is beyond the scope of this short educational article.

**Table 1:** Descriptive summary of some available genotype imputation panels.

Panel	N samples in panel	N variants	Types of variation available in the panel
TOPMed (Version r2)	97,256	308,107,085 genetic variants (of which 286,068,980 are single nucleotide variants) across the 22 autosomes and the X chromosome	single nucleotide variants and short indels
Haplotype Reference Consortium (Version r1.1 2016)	32,470	39,635,008	single nucleotide variants
1000 Genomes (Phase 3, version 5)	2,504	49,143,605	single nucleotide variants and short indels

## Tip 3: Use genetic association software that supports X chromosome testing

It is necessary to understand how conventional genetic association study software handles the X chromosome because different tools handle association testing on the X chromosome differently (**Table 2**). These differences can cause some missteps for researchers trying to include the X chromosome in their analyses for the first time. While there is no one recommended tool or approach for X chromosome association analysis, one study observed that in samples with a skewed male:female ratio, coding the male chromosome X genotypes as 0/2 (rather than 0/1) alleviated type 1 error [15]. Therefore, when male:female imbalance is present in an analysis dataset, coding of male genotypes on the X should be considered. We recommend implementing accepted genome-wide significant thresholds that account for the multiple testing inherent to genome-wide scans (for example, 5 × 10^−8^, which adjusts for 1 million independent tests) [16], rather than deriving X-specific significance thresholds.

**Table 2:** Non-comprehensive list of software for association testing, including chromosome X variants. Flags for single variant association testing are described.

Software	Base Considerations	Specific Flags	Description of Flags
PLINK v1.90 [17]	Treats X chromosome the same as autosomes unless a model is specified. NonPAR variants assigned to chr23 and PAR variants to chr25.	(1)--xchr-model (2)--merge-x	(1) Choose between 4 options: (i) exclude X chromosome, (ii) treat X chromosome the same as autosomes (and add sex as covariate), (iii) code male genotypes as 0/2 instead of 0/1, or (iv) add dosage–sex interaction to the model alongside sex as a covariate. (2) Merge PAR variants to be treated the same as the non-PAR variants.
PLINK v2.00 [18]	Treats X chromosome the same as autosomes unless a model is specified. Non-PAR variants assigned to chr23 and PAR variants to chr25.	(1)--xchr-model (2)--merge-par	(1) Choose between 3 options: (i) keep constant dosage, (ii) have different dosages of X chromosome depending on sex, or (iii) exclude the X chromosome. (2) Merge PAR variants to be treated the same as the non-PAR variants.
SAIGE v1.0.0 [19]	Treats X chromosome the same as autosomes.	(1)--X_ParRegion	Dosages of non-PAR regions of male chromosome X will be multiplied by 2.
REGENIE v3.3 [20]	Treats X chromosome the same as autosomes. Sex chromosomes will be collapsed into a single chromosome by default.	(1)--par-regions	Determine bounds for PAR1/PAR2 regions by using reference genome build code. Default is GRCh38.
BOLT-LMM v2.4.1 [21]	For the X chromosome, males should be coded as diploid, such that male genotypes are coded as 0/2 and female genotypes are coded as 0/1/2 (corresponding to a model of random X inactivation); PAR and non-PAR variants should be merged beforehand.	N/A	N/A
GCTA v1.94.2 [22]	NonPAR variants coded as 0/2 for males and 0/1/2 for females. Like PLINK, non-PAR variants assigned to chr23 and PAR variants to chr25.	--dc 1 or 0	Specify dosage compensation model for X chromosome variants where 1 = full (default) and 0 = no dosage compensation.
XWAS v3.0 [23]	XWAS adds X-specific functionalities on top of PLINK functions. For analysis of variants on the X-chromosome, XWAS removes all variants other than the ones on chromosome X, including variants of chromosome X’s PAR. Not to mention, X chromosome variants are filtered if they have significant differences in minor allele frequencies and/or missingness between male and female controls, with thresholds set in QC parameters.	(1)--xwas (2)--clayton-x (3)--strat-sex (4)--xchr-model (5)--run-all	(1) Proceed with XWAS unique commands, such as the ones below. (2) Test for association on the X chromosome using the method published by Clayton, 2008 [24]. (3) This flag will carry out sex-stratified analysis, and then combine the 2 results with options to modify male genotype output as either 0/1 or 0/2. (4) See (1) from PLINK v1.9. (5) Run Clayton’s test (see flag 2), sex-stratified test (see flag 3), and tests for difference in effect sizes or phenotypic variance in females vs. males

## Tip 4: Perform stratified association analyses for X chromosome variants

Stratified analyses carrying out association tests in individuals with XX karyotypes separately from those with XY karyotypes can be conducted to identify associations on the X (as well as for autosomal variants) that differ in magnitude, direction, or significance depending on sex chromosome karyotype. Tests of heterogeneity to identify genetic variants that exhibit significant association differences in the stratified association tests should also be carried out to quantity possible association differences. As sample sizes increase, individuals with sex-chromosome aneuploidy should be included in analyses, possibly stratified according to sex chromosome karyotype. However, stratifying study individuals into subgroups will reduce the total sample size for the association test. In datasets where statistical power is a concern, there are alternative methods to test for effect of sex, such as testing for genetic variant-by-sex interactions.

## Tip 5: At the minimum, assess biallelic single-nucleotide polymorphisms and variants as well as small insertions and deletions (indels) on chromosome X

Limited work has been done on investigating genetic variant–trait associations for more complex types of genetic variation on the X chromosome. Nevertheless, there is evidence of many types of genetic variation on the X chromosome contributing to complex disease risk, thus warranting additional investigations across traits and across types of variation (**Table 3**). When possible, expand to other variation types such as copy number and structural variants and include variants with more than 2 alleles (i.e., multi-allelic variants).

**Table 3:** Summary of selected types of genetic variation with known associations for complex human diseases or traits on chromosome X.

Type of variation	Brief description	Chr X example in the literature
Single-nucleotide polymorphism/variant (SNP/SNV)	A difference in a single nucleotide at a specific position in the DNA sequence	*GATA1* intronic SNP (X:48787974:G:A; GRCh38) associated with eosinophil count [25]
Insertion or deletion (indel)	Insertions or deletions of genetic sequence of up to 50 base pairs	*LINC01496* intronic deletion (X:51502424:GA/G; GRCh38) associated with prostate carcinoma [26]
Copy number variants (CNVs)	A type of structural variant with genomic segments of extra (duplications) or loss (deletions) of genetic sequence between 1,000 base pairs and 5 megabases [27]	Duplication in Xp22.33 region associated with urea [28]
Structural variants (SVs)	DNA rearrangements (e.g., inversions, insertions, deletions, translocations) involving at least 50 base pairs [29]	gnomAD SV provides a catalogue of SVs in the human genome, including on chromosome X [30]

## Tip 6: Consider implementing various statistical models to account for X inactivation

**Fig 1:**
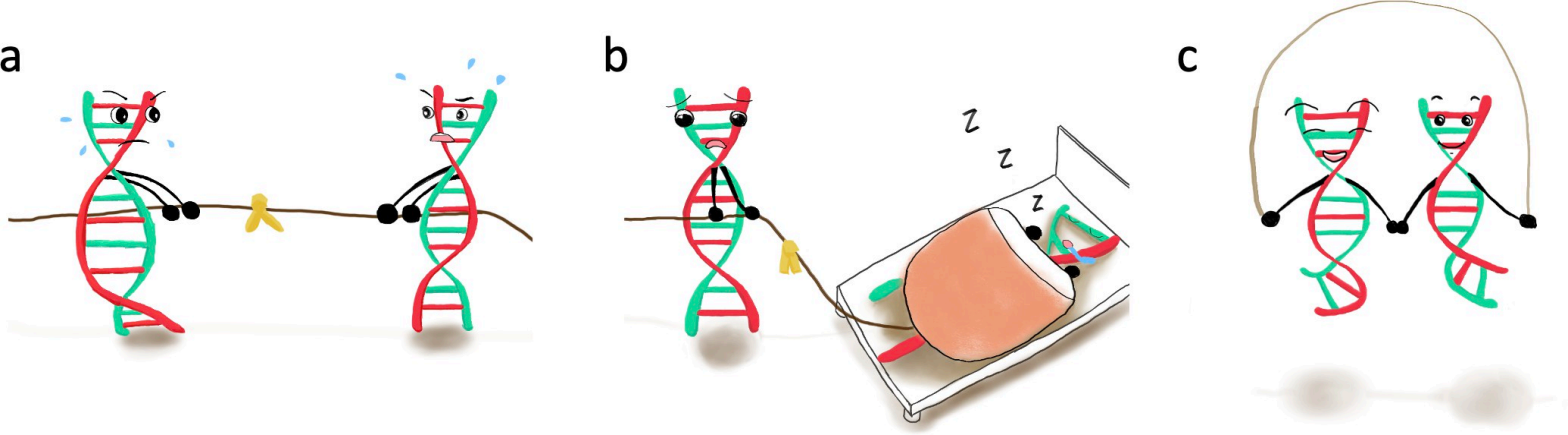
Representation of proposed X inactivation models in XX individuals. In random X inactivation, 50% of cells have 1 allele inactive, whereas the other 50% have the other allele inactive (**a**). In skewed (also known as nonrandom) inactivation, more than 50% of cells (say more than 75% or more extreme) have the same allele inactive (**b**). Finally, escape of X inactivation describes the scenario where both alleles are active in all cells (**c**).

In XX individuals, there can be compensatory mechanisms to reduce the dosage of gene expression in which one copy is subject to inactivation, resulting in only one copy of the gene being expressed (**Fig 1**). This biological factor must be considered while performing genetic association analyses on chromosome X. Statistical methods have been proposed to account for different inactivation models: random, skewed, or escape from inactivation [23,24,31,32]. PLINK [17,18] and XWAS [23] can model escape from inactivation or random inactivation, and there are models for skewed inactivation [31,33]. Given the lack of widely used standards and guidelines for association testing on the X chromosome, it can be useful to report association results from more than one applied method to further research in this direction using the most recent map of X inactivation across human tissues [34]. Applying and comparing association models accounting for different models of X inactivation for genetic variants on the X chromosome in XX individuals will be an important step forward to foster chromosome X association testing.

## Tip 7: Make your code and association summary statistics, including chromosome X results, publicly available

Data availability increases transparency and reproducibility and facilitates future research within the broader research community. Share your results with the broader community in a peer-reviewed publication describing your work. Sharing of summary statistics is commonplace and now required by many journals for publication of primary research articles. These summary statistics are used by the broader research community for a variety of tasks including replication of association signals, meta-analysis of cohorts in which the same or similar trait is measured, carrying out two-sample mendelian randomization, identification of variants to include in the development of a polygenic score and their corresponding weights, and many more downstream analyses. Not to mention, including chromosome X in summary statistics facilitates the inclusion of variants on this chromosome in these downstream analyses, furthering knowledge on the contribution of chromosome X variants to complex traits and diseases.

Code availability goes hand in hand with data availability in the call for open science. Making code available can provide other researchers with a starting point to facilitate the inclusion of X variants in their analyses. To this end, it is important to provide annotated code where the implemented steps are well documented. Guidelines described elsewhere should be followed to ensure code reproducibility that supports open science [35,36].

## Tip 8: Embrace the biological complexity and value of X chromosome analyses

By following these tips, we can all do our part in increasing the inclusion of chromosome X in association analyses and embracing our biological complexity through association analyses.

## Conclusions

Testing variants on the X chromosome is important for increasing understanding of this chromosome’s role in complex traits and diseases. Not to mention, making these results available to the broader community will facilitate downstream analyses including meta-analyses, the creation of polygenic risk scores and causal inference using mendelian randomization that incorporate chromosome X to advance the field. That being said, the inclusion of X chromosome genetic variants in association analyses is not a “one size fits all” approach. There is no single software or X inactivation model that will always give optimal results for every trait and in every study design. We encourage researchers to conduct further investigations to choose the best tool, resource, or method for their specific task using the information provided here as a starting point. All in all, addressing the challenges associated with chromosome X analyses will be crucial to foster future opportunities for scientific discovery related to topics that are not yet well understood, including, but not limited to, understanding haploid versus diploid states, sex chromosome aneuploidy states, and the biology of nonrandom X inactivation.
